# All-Optical Implementation of the Ant Colony Optimization Algorithm

**DOI:** 10.1038/srep26283

**Published:** 2016-05-25

**Authors:** Wenchao Hu, Kan Wu, Perry Ping Shum, Nikolay I. Zheludev, Cesare Soci

**Affiliations:** 1 Centre for Disruptive Photonic Technologies, TPI, Nanyang Technological University, 21 Nanyang Link, 637371, Singapore; 2 School of Electrical and Electronic Engineering, Nanyang Technological University, 50 Nanyang Avenue, 639798, SINGAPORE; 3 State Key Laboratory of Advanced Optical Communication Systems and Networks, Department of Electronic Engineering, Shanghai Jiao Tong University, 200240, China; 4 Optoelectronics Research Centre, University of Southampton, SO17 1BJ, UK

## Abstract

We report all-optical implementation of the optimization algorithm for the famous “ant colony” problem. Ant colonies progressively optimize pathway to food discovered by one of the ants through identifying the discovered route with volatile chemicals (pheromones) secreted on the way back from the food deposit. Mathematically this is an important example of graph optimization problem with dynamically changing parameters. Using an optical network with nonlinear waveguides to represent the graph and a feedback loop, we experimentally show that photons traveling through the network behave like ants that dynamically modify the environment to find the shortest pathway to any chosen point in the graph. This proof-of-principle demonstration illustrates how transient nonlinearity in the optical system can be exploited to tackle complex optimization problems directly, on the hardware level, which may be used for self-routing of optical signals in transparent communication networks and energy flow in photonic systems.

The science of stigmergy studies mechanisms of indirect communication mediated by the environment. Stigmergy was first observed in colonies of insects. For example, ants exchange information about the presence of food by secreting volatile chemicals (pheromones). Other ants of the colony are attracted by such pheromones, increasing the probability that foragers would find their way to the food. In this way ants collectively develop a complex network of trails connecting the nest to the foraging area, which can be regarded as a shared external memory for the entire colony^1^. In computer science, ant colony optimization (ACO) algorithms inspired by the natural stigmergic behavior of ants have been widely applied to complex optimization problems^2^. Numerical implementation of ACO algorithms was first proposed as multi-agent approach to complex combinatorial optimization, like the traveling salesman problem (TSP) and the nondeterministic polynomial (NP) problem of searching for the shortest closed path which visits all the destinations in a graph with given topology. Use of ACO algorithms in solving the TSP has proven to enhance convergence speed and improve likelihood to arrive at optimal solutions^3^,^4^,^5^. Meanwhile, ACO algorithms found application to routing problems in dynamic networks, where using ACO algorithms was shown to effectively solve the problem of routing and wavelength assignment (RWA) in optical networks^6^, optimization in cognitive radio networks^7^, and distributed control in communication networks^8^. ACO-based electronic circuits were also proposed for the development of bio-inspired hardware (BHW) able to change its architecture and behavior dynamically and autonomously while interacting with the environment^9^. Several hardware realizations of ACO algorithm based on field-programmable gate arrays (FPGAs) have been considered to search for optimal solution to a wide range of combinational optimization problems^10^,^11^ and to solve routing problems in networks^12^.

It has been recently proposed that *cognitive photonic networks* may allow implementing complex operations and mathematical functions beyond Boolean optical computing^13^,^14^,^15^. In this approach, information is typically stored by the network topology and operations result from light propagation and parallel distribution in the network, similar to electrical potential signals into the brain. Examples include neuro-inspired electro-optical reservoir computing^16^, which was successfully used for spoken digit recognition and nonlinear channel equalization^17^, and all-optical implementations of brain-inspired functions of various complexity, such as optical switching by coherent control of light^18^, solution of matrix inversion^19^ and solution of NP-complete problems using linear^20^ and non-linear^21^ optical networks.

In this paper, we discuss the realization of an all-optical stigmergic fiber network used to implement the ACO algorithm. We show that the presence of non-linear optical elements in the network induces the stigmergic behavior, enabling “cognitive” capabilities such as learning and plasticity. Optical implementation of ACO makes use of a nonlinear optical fiber network to represent the graph: by analogy between real and artificial ants represented by photons, here optical power corresponds to the number of ants, transient saturable absorption of erbium-doped fibers mimics the function of pheromone, and a reflective mirror is used to represent the food. ACO is demonstrated in two typical design schemes: in a tree-like scheme (Fig. 1(a,b)), where the path connecting the input and the mirror accumulates the largest optical power compared to other paths, and in a double-bridge scheme, with few alternative paths connecting the input to the mirror (Fig. 1(c,d)), where the optical output signal from the shortest path is reinforced as a function of time and optical input power. In both schemes, light transmission through the optical path connecting the input (nest) and the mirror (food) is dynamically reinforced, inducing preferential distribution of optical power (number of ants) in the shortest path to the mirror at steady state. This is analogue to the behavior of natural ant colonies, and sets the basis for all-optical implementation of stigmergy, in which transmission weights across different optical paths are altered upon training the network, the fundamental principle underlying plasticity of the brain.

## Method

**Figure 1:**
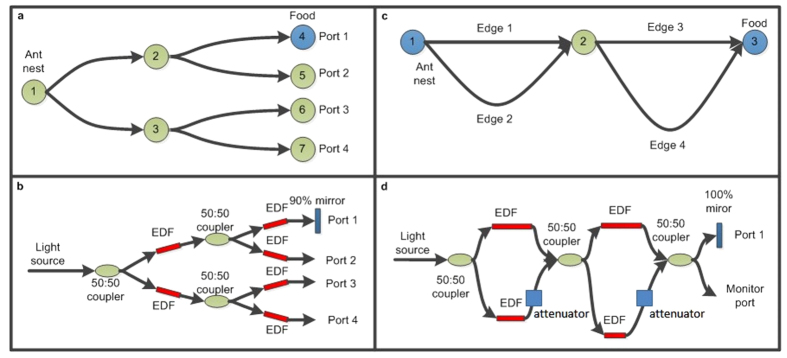
Graph topology and optical network realization of optical transmission reinforcement and ACO. (**a**) Topology of a two-layer binary tree graph with equal edges; the ant nest is located at node 1, and the food is located in node 4. (**b**) Optical network realization of the two-layer binary tree graph: light is injected from the “nest” and food is represented by a 90% reflective mirror placed at node 4 (output port 1). Nodes are realized by 50:50 couplers, and all edges comprise of SMF and EDF segments of identical lengths (60 cm and 8 cm, respectively). (**c**) Topology of a two-layer double bridge graph; the ant nest is located at node 1, and the food is stored at node 3. Route of edges 1 + 3 is the shortest route (edge 1 = edge 3 < edge 2 < edge 4). (**d**) Optical network implementation of the two-layer double bridge graph: the network input corresponds to the ant nest and a 100% reflective mirror mimics the food. Nodes are realized by with 50:50 coupler and edges built with SMFs containing EDF segments. The length of SMF is proportional to the length of the corresponding graph edge, while the length of EDF segments is inversely proportional to the length of the graph edges. Attenuators are used to compensate for linear losses, ensuring that all paths have identical low-power transmission.

Mathematical graph problems can be directly mapped onto optical networks with corresponding topology and node coupling properties^19^,^20^,^22^,^23^. In the case of ACO, ant activity is simulated by light propagating and distributing through a telecom fiber network at 1.55 μm. Low-loss optical single mode fibers are used to construct the network, where the length of optical fibers is proportional to the edges of the target graph. For the tree-like scheme in Fig. 1a, both 1.55 μm continuous wave (CW) laser and pulsed laser are used as the light source to mimic the ant nest. The number of ants is represented by the average optical power for the CW input, and by the pulse energy in the case of pulsed input. Conversely, the double-bridge scheme in Fig. 1c uses a pulsed laser source to identify the different propagation paths from the timing of the output pulses; the number of ants is then represented by the optical pulse energy. Food in the ACO problem is physically realized by a reflective mirror placed at a given output port of the optical network: back propagating light reflected by the mirror acts as ants returning to the nest after finding the food. Finally, the signaling role of pheromone is simulated by saturable absorption properties of Erbium-doped fiber (EDF) segments inserted into the network^24^. Pheromone is a chemical secreted by ants to pass information on previously visited paths to succeeding ants. It is released by ants that find food on their way back to the nest, attracting other ants toward the same path. By this stigmergic communication, paths that lead to food are chosen more frequently than others, increasing foraging success of the entire colony over time. A key characteristic of pheromone is being volatile: its slow dissipation allows the colony not only to find a way to the food, but also to identify the optimal (shortest) path to it. Pheromone characteristics are here reproduced by nonlinear properties of EDFs: transmission of un-pumped EDFs is power dependent (increases at high input powers due to saturation effects). Moreover, saturable absorption decays over time due to spontaneous emission in the EDF, analogue to dissipation of the pheromone. To reproduce path reinforcement in ACO, EDF segments are inserted into the SMF network, so that the optical feedback provided by the mirror in the food position induces the progressive increase of optical fluency along the path connecting the input and the mirror ports. Simulations are also performed by tracing the light propagating in the fiber network in which the saturable absorption of EDF is modeled as *T* = 1 − *l*_0_/(1 + *P*/*P*_*0*_), where *T* is the transmission of EDF, *l*_0_ is the loss of EDF at low input power, *P* is the input power and *P*_0_ is the saturation power. The values of *l*_0_ and *P*_0_ are obtained by fitting the experimental data. Iteration is used until the simulation reaches a steady state.

## Results and Discussion

The first step to implement all-optical ACO algorithms is to establish the parameter field in which optical path reinforcement (plasticity) can be achieved. As a proof-of-concept demonstration, optical path reinforcement is first implemented in a simple two-layer full binary tree scheme, where all possible paths have equal length but only one path connects the ant nest (input port) to the food (mirror at one output port), as shown in Fig. 1a. The actual realization of the graph in fiber network is shown in Fig. 1b: light is injected from node 1 (represents ants leaving the nest in search for food), and a 90% mirror is placed at output port 1 (acts as the food source). Nodes 1–3 are realized using 50:50 fiber couplers. In this case all edges between nodes comprise of SMF and EDF segments of identical lengths (60 cm and 8 cm, respectively). When light is injected into the network, it is equally distributed among all edges by the 50:50 couplers. This corresponds to ants initially exploring all possible paths with equal probability. Meanwhile, light propagation increases transmission of the EDF segments uniformly. 90% mirror in port 1 increases optical power in the path connecting nodes 1–2–4, further bleaching the corresponding EDF segments and increasing light transmission along the one path from “nest” to “food”. Overall the back propagating light beam provides positive feedback on light transmission, alike an increase of pheromone in the path. The optical power monitored at output port 1 is then expected to reach a steady-state value higher than the one monitored at output ports 2–4 (nodes 5–7), similar to an increase of the number of ants treading the foraging path. Note that, in experiments, only 10% of the actual optical power is measured at output port 1 due to the 90% reflection of the mirror, therefore intensity values are multiplied by a factor of 10 for direct comparison with other output ports.

**Figure 2:**
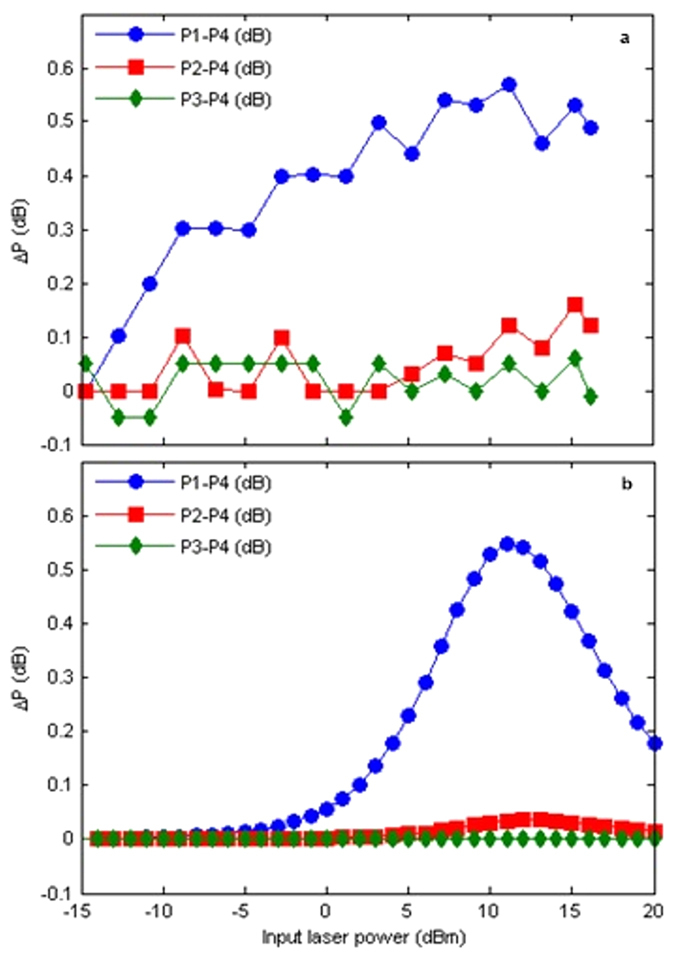
Optical path reinforcement in a two-layer binary tree network with continuous wave light input. The relative optical power measured at output ports 1–3 is plotted using port 4 (the farthest away from the mirror) as a reference. Experimental results (**a**) match well the simulation results (**b**). Output power difference between port 1 and port 4 is as high as 11% for input power of ~11 dBm. Meanwhile, the difference between output from port 2 and port 4 can reach 4%, while there is nearly no difference between port 3 and port 4, irrespectively of input power.

Evidence of optical path reinforcement in the two-layer binary tree graph with CW light input is given in Fig. 2. Here the output power from the most distant port to the mirror (port 4) is used as reference, considering the difference between the output energy form ports 1–3 and port 4. From the experimental measurements in Fig. 2a it can be seen that the output power at the port with the mirror (port 1) is higher than the other three ports. The relative increase in output power at port 1 depends on input power, with a maximum when input power is ~11 dBm. This is due to the fact that, upon reaching deep saturation of the EDF segments in the path with the mirror, further increase of optical input power can only increase transmittance in the other paths, thus reducing the relative difference measured at port 1. In this case, maximum reinforcement obtained at 11 dBm input power is 0.55 dB, or 11%, in good agreement with the simulation results shown in Fig. 2b. Further increase of EDF length can slightly increase the maximum power difference ∆*P* and will shift the peak of ∆*P* (blue curves in Fig. 2a,b) to a higher input power level.

**Figure 3:**
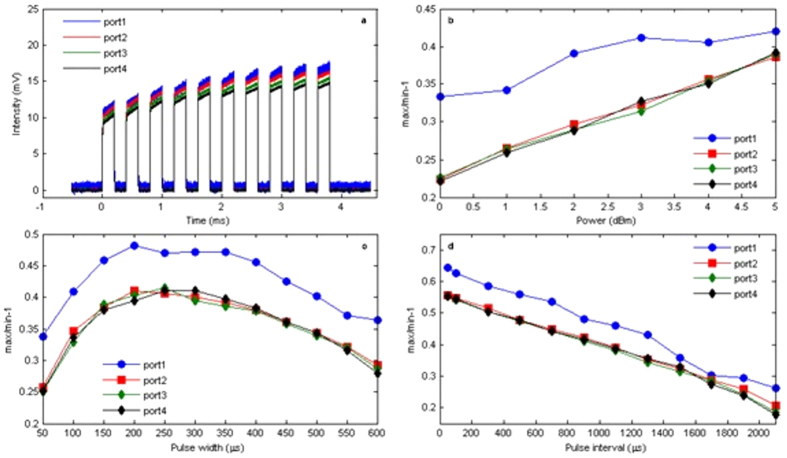
Optical transmission reinforcement in a two-layer binary tree network with pulsed laser input. (**a**) Pulse waveforms measured at the four output ports upon injection of a ten-pulse bundle every 10 ms period (individual pulse width and interval are 200 μs). The vertical axis is the voltage after the photodetector of the output optical signal. The cumulative increase of optical transmission upon arrival of successive pulses is quantified by the relative increase of output intensity between the first and the last pulses (max/min − 1) as a function of input pulse energy (**b**), input pulse width (**c**) and input pulse interval (**d**).

The dynamics of path reinforcement was studied using pulsed laser input in the two-layer binary tree graph (Fig. 3), where additional input parameters such as pulse energy, pulse width and repetition rate can be tuned independently. The pulse train is generated by an acousto optical modulator (AOM) Input pulses were chosen to maximize visibility of reinforcement at output port 1, with 200 μs pulse width, 5 kHz repetition rate and a 10-pulse sequence injected into the graph every 10 ms (this time was found to be long enough to allow full relaxation to the original state between consecutive bundles). The corresponding output waveforms monitored by a real-time oscilloscope are displayed in Fig. 3a, showing an overall increase of light transmission through the network due to saturation of the EDF segments upon injection of successive pulses, and relative increase of light intensity in the path containing the mirror, as already observed in the CW case. The dependence of relative output intensity between the first and the last pulse of a train (max/min − 1) on input pulse parameters (i.e. pulse intensity, width, and repetition rate) is shown in Fig. 3b–d. The higher the input pulse energy, the larger the relative increase of output intensities (Fig. 3b): in this regime (a train of ten pulses injected into the graph every 10 ms, with pulse repetition rate of 5 kHz), the output at the port with the mirror (port 1) shows the largest reinforcement of optical transmission, although it shows signs of saturation at the highest input powers investigated. For a given repetition rate of the train (e.g. 5 kHz), increasing pulse width increases the overall optical power injected into the network, with similar effects induced by CW light power (Fig. 3c): the relative output intensities first increase with the pulse width and reach a maximum around 250 μs, and then drop due to saturation of the EDF segments. This corresponds to a regime where the energy of the first pulse of the train exceeds saturation threshold of the EDF segments in the path with the mirror, so that further increase of pulse width (optical power) reduces cumulative effects of the pulse train on EDFs saturable absorption and enhancement of light transmission. All four output ports show similar trends, with the port with the mirror (port 1) having the largest reinforcement. Similarly, increasing repetition rate of the pulse train (while keeping width constant at 200 μs) increases the average input power (Fig. 3d): overall light transmission increase at high repetition rates and port 1 maintains the largest reinforcement of optical transmission. This is analogue to what observed in Fig. 3b, but with no sign of saturation at high repetition rates.

These simple experiments prove progressive reinforcement of optical transmission in the path connecting the input port (ant nest) to the mirror (food), which can be arbitrarily placed in any of the equivalent outputs. Overall this can be seen as a learning process enabled by the inherent plasticity of the nonlinear fiber network, where the network reconfigures itself to enhance optical power (number of ants) standing between the input (nest) and the mirror (food). From a mathematical viewpoint, the optical network converges to a physical stationary solution that indicates a path connecting the input node to an arbitrary outer node of the graph. In the following we show that properly designed nonlinear fiber networks can also converge to the optimal solution (i.e. the minimal path) between the input and a chosen node of the graph, as required by ACO algorithms.

ACO is demonstrated in a two-layer double-bridge graph where multiple paths of different lengths connect the ant nest with the food source, as shown in Fig. 1c. The graph comprises of four edges of various lengths (for simplicity, edge 1 and edge 3 are chosen to be equal). The ant nest is allocated at node 1 and food is stored in node 3, so that there are four possible paths to reach the food from the nest (edges 1 + 3, 2 + 3, 1 + 4 and 2 + 4), of which edges 1 + 3 is the shortest. The actual realization of the graph is shown in Fig. 1d. A pulsed laser source is injected into node 1 and split equally by a first 50:50 coupler. Node 2 is formed by a second 50:50 coupler, and a third 50:50 coupler is used to place a 100% mirror in node 3 and monitor the output simultaneously. The length of EDF segments in each path of the network is chosen to be inversely proportional to the edges of the target graph. The actual lengths of optical fiber used in our realization were: 1 m SMF and 20 cm EDF in edge 1, 1.6 m SMF and 12.5 cm EDF in edge 2, 1 m SMF and 20 cm EDF in edge 3, and 2.2 m SMF and 9.09 cm EDF in edge 4. Two variable attenuators used to compensate for linear insertion losses in the different optical paths are added in edges 2 and 4. Constant attenuation values are determined at the lowest input power to ensure that all the paths have identical linear transmission. Since after compensation of linear losses longer EDFs yield higher transmission at a given optical power, shorter edges of the network are then reinforced more effectively than longer branches when more light (ants) passes through it; conversely, the shortest (optimal) path between the input (nest) and the mirror (food) is expected to attain the highest transmission and contain the highest optical power (number of ants) at steady state.

**Figure 4:**
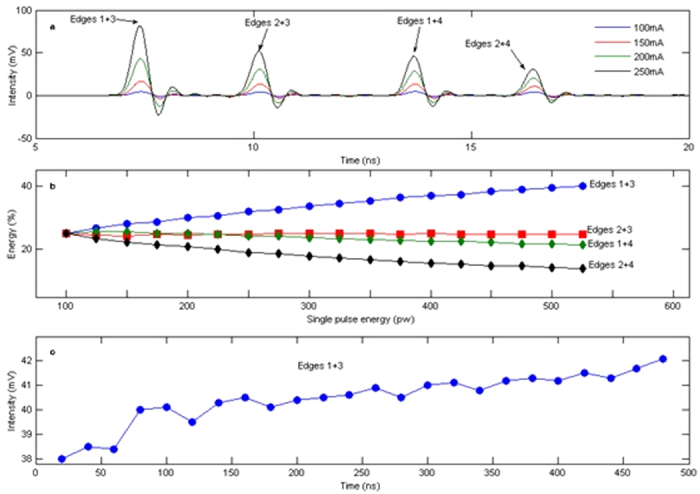
Optical implementation of ACO in a two-layer double bridge network. (**a**) Output waveforms measured upon injection of a laser pulse, at different input powers. The four output pulses appearing at different time delays correspond to the four possible routes between the input and the output nodes in the network, and assigned based on the total length of the corresponding path edges. (**b**) Proportion of energy flowing into the four possible paths obtained by calculation of output pulse intensities, as a function of input pulse power. (**c**) Dynamics of optical transmission reinforcement in the shortest path of the network (edges 1 + 3) upon injection of bursts of 24 pulses spaced by 20 ms.

Experimental results obtained in the two-layer double bridge network are shown in Fig. 4. With an input pulse obtained by a femtosecond pulsed laser (repetition rate of 50 MHz), pulses travelling through the four different paths of the network can be distinguished by monitoring the time delay of the output. Fig. 4a show the waveforms measured at the output port for different input average power. The four pulses at different time delays correspond to propagation through edges 1 + 3, 2 + 3, 1 + 4, and 2 + 4, as determined by the retardation induced by optical fibers of the given lengths. It can be seen that for higher average input power (the unit used in the graph indicates current of the laser pump diode, which is proportional to emission power), the output intensity of all pulses increases, and the pulse traveling through edges 1 + 3 (the shortest path between input and mirror) is consistently the highest. The proportion of energy flowing into the four possible paths is calculated of the output pulses, and its dependence on input power is shown in Fig. 4b. At low input power, energy is distributed equally into the four equivalent paths (25% in each of the paths); however, at high input power, transmission through edges 1 + 3 increases, and the corresponding path accumulates up to 40% of the total energy injected into the network, mainly at the expenses of edges 2 + 4 (in which energy decreases from 25% to 13%), and edges 1 + 4 (in which energy drops by nearly 3%). The fractional energy in edges 2 + 3 remains almost unchanged. To achieve these results, input intensity was properly chosen to induce different saturation state of the EDF segments in the available paths. Specifically, edges 1 + 3 are far from saturation, edges 2 + 4 are completely saturated, while edges 1 + 4 and edges 2 + 3 are near saturation. These conditions can be obtained simultaneously since the length of the EDF segments was set to be inversely proportional to the physical length of the edges of the graph. Finally, to investigate the transient dynamics of optical transmission reinforcement in the shortest path a burst pulse sequence (24 pulses per burst, 20 ms spacing between bursts) was injected into the network, and the average output intensity corresponding to the bursts treading edges 1 + 3 was monitored over time (Fig. 4c). It can be seen that with these input conditions the average output intensity increases by nearly 10% between the first and last injected pulses, consistent with the cumulative saturation of EDF segments competing with natural decay of saturable absorption on a time scale of milliseconds. As mentioned, matching the input beam parameters to the threshold and decay time constant of the network nonlinearity is necessary to implement ACO and avoid convergence to local minima; although the proposed scheme for optical implementation of ACO algorithm could be readily transferred to integrated silicon photonics or plasmonic networks to solve problems of greater size and complexity, such constraints should be taken into account when designing nonlinear optical networks based on different nonlinear processes.

## Conclusions

In conclusion, we demonstrated an all-optical experimental primer of a nonlinear stigmergic fiber network that solves the ACO algorithm. As a demonstration of principle we have shown that, in a two-layer tree graph, selective reinforcement of the optical transmission can be achieved in a relatively large space of input beam parameters, operating with either CW or pulsed excitation. Actual ACO was achieved in a two-layer double bridge graph, where the network is shown to converge autonomously to the minimal-path solution within few milliseconds, and with visibility greater than 10%. We propose that self-learning capabilities of nonlinear optical networks, combined with their possible implementation on reconfigurable integrated silicon photonics or plasmonic platforms, may be used to implement optical computing schemes beyond Boolean logic. Even for limited sizes, all-optical “cognitive photonic networks” have already proven viable to solve computationally hard problems, such as NP complete problems^20^,^21^ and quantum simulators^25^,^26^. In addition, implementation of ACO at the hardware level may find practical applications like optical signal routing in dynamic networks, self-reconfiguring optical networks, and optical image processing devices, which could be faster and more energy-efficient than existing electronic-based solutions.

### Data Availability

Following a period of embargo, the data from this paper can be obtained from the University of Southampton ePrints research repository, http://dx.doi.org/10.5258/SOTON/394237.

## Additional Information

**How to cite this article**: Hu, W. *et al*. All-Optical Implementation of the Ant Colony Optimization Algorithm. *Sci. Rep*. **6**, 26283; doi: 10.1038/srep26283 (2016).
